# The Hospital World

**Published:** 1910-10-08

**Authors:** 


### The Hospital World.

### Elections and Appointments.

The Duke of Rutland has been re-elected President of the Ilkeston Hospital.

### Personalia.

His Majesty the King has consented to become patron of St. Bartholomew's Hospital.

Her Majesty the Queen has become patroness of the Aberdeen Royal Hospital for Sick Children.

Mrs. Wilson Noble will be "At Home" to members of the Berkshire District of the League of Mercy, in the Barkham Club Room, at 3 o'clock, and afterwards at Barkham Manor, on Saturday, October 15.

Mr. John Henderson, one of the best-known merchants in Liverpool, has died at the age of 74. As a member of the Liverpool City Council, in which his interest in corporation finance made him an important factor, even after his retirement, and as a Liberal he was a great local figure; but it was the generous way in which he expended his wealth by which, after all, perhaps he was most respected and known. One of the institutions particularly dear to him was the Liverpool Royal Infirmary, which owes him much in many directions, personal as well as financial, and which will cherish his memory. District nursing was another matter on which his enthusiasm exerted itself, and the Liverpool Queen Victoria District Nursing Association has lost in his death a friendly critic and firm supporter. In the North of England especially the mantle of public spirit and benevolence falls on succeeding generations, but few have carried it more finely than Mr. John Henderson. A strong link with the past has snapped at his decease.

Our account of Sir George Bartley, K.C.B., whose death last month removed one of the most distinguished Vice-Presidents of the Hospital Saturday Fund, has been held over unavoidably until to-day. Known to the world as the founder of the National Penny Bank, the pioneer of the thrift movement among the poor, it is apparent how valuable his knowledge and experience were to a fund which above everything else has to support charitable institutions without injuring the personal respect of individuals. It used to be a claim of his that he was the wicked man who started the idea of old-age pensions in 1871. It was characteristic of the disinterestedness with which Sir George Bartley carried on his work that he spared no pains to make the success of the Penny Bank independent of any one life and personality. A company was formed, and in the corporate life of that the Bank lives on, with its 160,000 depositors. It was at the Hospital Saturday Fund's annual dinner two years ago last February 1 that his great speech on "Thrift" made his chairmanship of the evening an occasion to be remembered in the annals of the Fund.

